# Clinical characteristics of male prolactinoma patients mainly presenting with severe obesity and the metabolic response to dopamine agonist therapy

**DOI:** 10.3389/fendo.2023.1285477

**Published:** 2023-11-29

**Authors:** Linjie Wang, Xiaojing Wang, Fengying Gong, Hui Pan, Huijuan Zhu

**Affiliations:** ^1^ Department of Endocrinology, Key Laboratory of Endocrinology of National Health Commission, Peking Union Medical College Hospital, Peking Union Medical College, Chinese Academy of Medical Sciences, Beijing, China; ^2^ Shanxi Yuncheng Central Hospital, Yuncheng, China

**Keywords:** Prolactinoma, obesity, male, dopamine agonist, metabolic response

## Abstract

**Objective:**

To summarize the clinical characteristics of 4 male prolactinoma patients with severe obesity.

**Methods:**

The clinical data of all the patients were retrospectively analyzed.

**Results:**

All the patients visited our hospital for severe obesity at the age of 16-30 years old with their body mass index (BMI) of 37.9-55.9 kg/m^2^. All the patients were obese since childhood, even at birth. Hyperprolactinemia (72.3-273.0 ng/ml) was found during the etiological screening of obesity and MRI revealed pituitary adenomas. Additionally, all of them had multiple obesity related complications, such as hyperinsulinemia and dyslipidemia. Treatment of dopamine agonists (DAs) effectively normalized their prolactin level and the pituitary MRI reexamination after 6 months of DAs treatment showed the shrinkage of the pituitary adenomas in 3 patients. Their weight also decreased in different degrees (2.70~19.03% lower than the baseline) with improved metabolic profiles.

**Conclusion:**

Serum prolactin level should be screened in obese patients, especially those with severe obesity.

## Introduction

Obesity is an overwhelming prevalent chronic metabolic disease caused by a variety of factors. Previous studies have demonstrated that hyperprolactinemia, especially prolactinoma, can lead to obesity (1). Meanwhile, obesity has also been proved to be related to dysfunction of dopaminergic pathways (2). Here we summarized the clinical characteristics of 4 male prolactinoma patients, mainly presenting with severe obesity, which might provide us with further insights into the relationship between obesity and hyperprolactinemia.

## Methods

Clinical data from 4 male prolactinoma patients with early-onset severe obesity in the endocrinology department of a tertiary medical center Peking Union Medical College Hospital of China from September 2016 to August 2022 were retrospectively analyzed.

## Results

### Baseline clinical characteristics

As shown in **Table 1**, all of the 4 patients (Case 1 to 4) visited our hospital due to severe obesity. Their age at the first visit was 16-30 years old, and their body mass index (BMI) was 37.9-55.9 kg/m^2^. All the patients had been obese since childhood, and the birth weight of 3 patients was over 4 kilograms. Hyperprolactinemia (72.3-273.0 ng/ml) was found during the aetiological screening of obesity. Further inquiry revealed that they denied any medical history of antipsychotics and gastric motility drugs. All of them denied delayed puberty. They had no complaints of headache or vision impairment. 3 patients reported hypolibido and erectile dysfunction. Physical examination showed that all of them had gynaecomastia without galactorrhea and their testes were normal in size. The evaluation of other anterior pituitary function showed that they all had hypogonadotropic hypogonadism, and 2 of them had decreased insulin like growth factor 1 (IGF1). None of the patients had secondary hypothyroidism or adrenal insufficiency. MRI revealed pituitary adenomas (the maximum diameter ranged from 9 to 17 mm). Therefore, prolactinoma was suspected. The levels of PTH, serum calcium, glucagon and gastrin were measured, which were all in normal range excluding the clinical diagnosis of multiple endocrine neoplasia type 1. No genetic screenings of *AIP* or *MEN1* mutations were performed.

**Table 1:** The clinical features of 4 male prolactinoma patients mainly presenting with early-onset severe obesity.

Variables	Case 1	Case 2	Case 3	Case 4
clinical features at first visit				
Visit age (years)	20	30	26	16
Birth weight (kg)	4.2	4.4	3.0	4.1
Onset time of obesity (years)	since born	since born	6	since born
Time of obesity duration (years)	20	30	20	16
Gynaecomastia	Y	Y	Y	Y
Galactorrhea	N	N	N	N
Sexual dysfunction	Y	Y	Y	N
Headache	N	N	N	N
Vision Impaired	N	N	N	N
Weight (kg)	176.8	185.0	109.4	176
Height (cm)	180.0	182.0	170.0	191.0
BMI(kg/m^2^)	54.6	55.9	37.9	48.2
Waist circumference (cm)	158.0	154.0	117.1	142.5
Blood pressure (mmHg)	150/100	160/95	140/90	120/78
PRL (normal range 2.6-13.1 ng/ml)	273.0	93.4	196.0	72.3
LH (mIU/L)	1.93	3.44	2.77	5.15
FSH (mIU/L)	1.04	4.72	4.44	5.11
T (normal range 1.75-7.81 ng/ml)	0.91	1.92	1.26	1.49
Estradiol (normal range <47 pg/ml)	14	30	27	18
IGF-1 (ng/ml)	188	53	169	198
IGF-1/LLN	1.34	0.54	1.46	0.88
Morning plasma ACTH (0-46 pg/ml)	68.8	/	37.6	23.9
Morning serum cortisol (4.0-22.3μg/dl)	15.70	18.23	18.93	19.1
Overnight low dose dexamethasone suppression test	suppressed	suppressed	suppressed	/
TSH (0.38-4.34μIU/ml)	3.038	3.326	3.62	2.359
FT_4_ (0.81-1.89 ng/dl)	1.040	1.124	1.192	1.11
HbA1c (%)	5.3	6.4	5.2	6.0
OGTT-FBG (mmol/L)	5.7	5.5	5.7	5.1
OGTT-2hPBG (mmol/L)	6.7	5.7	8.5	5.5
Fasting INS (IU/ml)	15.65	91.64	35.74	56.9
OGTT-INS_max_ (IU/ml)	>300	>300	248.69	>300
ALT (U/L)	94	142	76	245
AST (U/L)	43	80	36	84
UA (umol/L)	602	353	493	487
TC (mmol/L)	4.67	5.44	4.99	4.38
LDL-c (mmol/L)	3.53	3.92	3.46	3.03
HDL-c (mmol/L)	0.68	0.83	1.02	0.94
TG (mmol/L)	1.19	1.5	2.18	1.20
abdominal ultrasonography	no obvious abnormality	fatty liver	fatty liver	severe fatty liver
Maximum diameter of PAs (mm)	17	9	10.6	9
Treatment and follow-up				
Duration of follow-up (months)	44	24	60	14
Treatment choice of prolactinoma	drug	drug	drug	drug
Type of DAs	bromocriptine→ cabergoline	bromocriptine	bromocriptine	bromocriptine
Maximum dose of bromocriptine (mg/d)	15	7.5	5	5
Maximum dose of carbergoline (mg/w)	2	–	–	–
Minimum PRL level during follow-up (ng/ml)	7.78	4.86	5.3	15.5
Maximum T level during follow-up (ng/ml)	3.47	3.14	3.63	2.65
Maximum estradiol level during follow-up (pg/ml)	57	90.36	75.78	24
Maximum diameter of PA half a year after treatment(mm)	14	–	7.5	8.4
Other concomitant drugs	metformin 1.5 g/d amlodipine 5 mg/d	metformin 1.5 g/d nifedipine 30 mg/d bisoprolol 5 mg/d	metformin 1.5 g/d losartan 50 mg/d	metformin 1.5 g/d
Last follow-up				
DAs treatment	cabergoline 2mg/w	bromocriptine 2.5 mg/d	bromocriptine 1.875 mg/d	bromocriptine 3.75mg/d
Weight (Kg)	169	180	95	142.5
Weight loss percent from baseline (%)	4.41	2.70	13.63	19.03
BMI (kg/m^2^)	52.2	54.3	32.8	39.1
Blood pressure (mmHg)	120/80	130/70	120/90	/
PRL (ng/ml)	30.9	11	10.8	15.5
FBG (mmol/L)	5.1	5.4	5.2	5.33
Fasting INS (μIU/ml)	15.18	62.14	9.1	26.1
HbA1c (%)	5.4	5.3	5.0	5.5
ALT (U/L)	15	57	25	78
UA (umol/L)	521	531	564	415
TC (mmol/L)	4.58	5.01	5.12	4.22
LDL-c (mmol/L)	3.1	3.77	3.65	2.55
HDL-c (mmol/L)	–	0.94	0.94	0.88
TG (mmol/L)	0.77	1.07	1.69	1.78

BMI, body mass index; Y, yes; N, no; PA, pituitary adenoma; FSH, follicle stimulating hormone; LH, luteinizing hormone; T, testosterone; PRL, prolactin; IGF1, insulin like growth factor 1; LLN, lower limits of normal; ACTH, adrenocorticotropic hormone; TSH, thyroid-stimulating hormone; FT4, free tetraiodothyronine; OGTT, oral glucose tolerance test; FBG, fasting blood-glucose; PBG, postprandial blood glucose; INS, insulin; ALT, alanine aminotransferase; UA, uric acid; TC, total cholesterol; LDL, low density lipoprotein; HDL, high density lipoprotein; TG, triglyceride.

All the patients were evaluated for obesity related complications: hyperinsulinemia was found in all of them and Case 3 had impaired glucose tolerance; 3 patients had dyslipidemia, including elevated low density lipoprotein (LDL-c) and triglyceride (TG), and decreased high density lipoprotein (HDL-c); All of them had abnormal liver function and fatty liver was found in 3 patients by abdominal ultrasound. 3 patients had hypertension; 3 patients had hyperuricemia.

### Treatment and follow-up

The above patients were followed up for 14 ~ 44 months in our center.

Dopamine agonists (DAs) were used to treat their hyperprolactinemia. Case 1 was initially treated with bromocriptine. The drug dose was gradually increased to 15mg/d according to his prolactin (PRL) level. However, his prolactin level was still significantly increased at about 100ng/ml, suggesting the resistance for bromocriptine. Cabergoline was then used with the maximum dose of 2mg/w, resulting in a PRL reduction to below 30ng/ml. Cases 2 to 4 were treated with bromocriptine with the maximum dose of 5 to 7.5 mg/d, and their PRL level were successfully controlled within 20 ng/ml. In Case 1, 3 and 4, after 6 months of DAs treatment, the pituitary MRI reexamination showed that the pituitary adenomas had shrunk compared to before. During the follow-up, the testosterone levels of all patients were significantly higher than the baseline, and their erection dysfunction was improved. The monitoring of sex hormones revealed that the estradiol in Case 1 to 3 increased intermittently, with the maximum level of 57 to 90.36 pg/ml.

In addition, lifestyle guidance for obesity was given to all the patients. Metformin and antihypertensive drugs were administered according to the complications (**Table 1**). Their weight decreased in different degrees (2.70 to 19.03% lower than the baseline) during the following-up. At the same time, fasting insulin and liver function of all the patients were improved.

## Discussion

Another 3 adult male prolactinoma patients with severe obesity were reported previously (3–5) (as shown in **Table 2**). Their age at diagnosis of prolactinomas was 24-39 years old, with their BMI of 64.4, 47.09 and 45.67 kg/m^2^, respectively. 2 of them mentioned their obesity onset age of 19 and 17 years old. Along with our patients, they also got weight loss and improvement of their metabolic abnormality after DAs, bariatric surgery and other treatments.

**Table 2:** Main clinical characteristics of 3 male prolactinoma patients with severe obesity in the literature.

Variables	Case 1^[3]^	Case 2^[4]^	Case 3^[5]^
Age at diagnosis of prolactinoma (years)	39	24	30
Onset age of obesity (years)	19	17	NR
Duration of obesity (years)	20	7	NR
BMI(kg/m^2^)	64.40	47.09	45.67
PRL (ng/ml)	82.6	73.14	315.0
LH (mIU/L)	1.8	NA	1.12
FSH (mIU/L)	1.2	NA	1.4
T (ng/ml)	0.75	0.87	1.1
Maximum diameter of PAs (mm)	10.0	6.0	17.0

NR, not recorded; BMI, body mass index; PRL, prolactin; LH, luteinizing hormone; FSH, follicle stimulating hormone; T, testosterone; PA, pituitary adenoma.

PRL has been recognized as a regulatory factor of energy homeostasis during physiological and pathophysiological conditions, such as increasing leptin synthesis and secretion, permitting the circadian variation in lipogenic responsiveness (6–9). Previous animal and clinical studies have shown that hyperprolactinemia can cause obesity and related metabolic abnormalities. In the female mice lacking dopamine D2 receptors in lactotropes, long-term chronic hyperprolactinemia was found to increase the expression of the orexigenic genes, such as neuropeptide Y, in the hypothalamic arcuate nucleus and ventromedial nucleus, resulting in obvious weight gain and leptin resistance from the age of 5-10 months (10). Moreover, severe hyperprolactinemia was observed to promote brown adipose tissue whitening and exacerbate high-fat-diet-induced energy imbalance (11). On the contrary, in the mice lacking prolactin receptors, their beige differentiation of adipose depots was found to protect against high-fat-diet-induced obesity (12). In clinical studies, it was observed that prolactinoma patients had higher BMI than the general population, and the BMI of male patients increased more significantly (1). Additionally, the average BMI of patients with macroprolactinomas was significantly higher than that of patients with nonfunctioning pituitary macroadenomas (13).

Obesity can lead to dysfunction of dopamine related pathways: obesity was found to affect the availability of dopamine transporter in the midbrain striatum (14), lower forebrain dopamine levels (15, 16); Additionally, the level of dopamine D2 receptor in obese patients was lower, and its availability was also decreased (17–19).

Till now, DA is still selected as the first-line treatment for most prolactinomas (20). Mirjana Doknic,et al had reported that bromocriptine, by increasing dopaminergic tone, could influence body weight and likely body composition by mechanisms in addition to reducing hyperprolactinemia in prolatinoma patients (21).Ezrokhi M et al. revealed that timed daily DA treatment improved hypothalamic and neuroendocrine pathologies associated with metabolic syndrome in SHR rats, which coupled to a transformation of liver metabolism potentiating a reduction of elevated lipogenic and gluconeogenic capacity (22). Therefore, DAs therapy for the prolactinoma patients might bring additional metabolic benefits beyond simply reducing hyperprolactinemia.

In conclusion, this paper describes the clinical characteristics of 4 male prolactinoma patients with severe obesity as the main clinical manifestation. Hyperprolactinemia/prolactinoma can cause and aggravate obesity through a variety of ways. Serum prolactin level should be screened in obese patients, especially those with long-term and severe obesity, in order to avoid miss-diagnosis of hyperprolactinemia.

## Data availability statement

The raw data supporting the conclusions of this article will be made available by the authors, without undue reservation.

## Ethics statement

Ethical approval was not required for the study involving humans in accordance with the local legislation and institutional requirements. Written informed consent to participate in this study was not required from the participants or the participants’ legal guardians/next of kin in accordance with the national legislation and the institutional requirements. Written informed consent was obtained from the individual(s), and minor(s)’ legal guardian/next of kin, for the publication of any potentially identifiable images or data included in this article.

## Author contributions

LW: writing – original draft. XW: writing – review & editing. FG: writing – review & editing. HP: writing – review & editing. ZH: writing – review & editing.
